# The interaction between motor simulation and spatial perspective-taking in action language: a cross-cultural study

**DOI:** 10.3758/s13421-023-01427-1

**Published:** 2023-05-19

**Authors:** Cosimo Tuena, Daniele Di Lernia, Claudia Rodella, Francesca Bellinzona, Giuseppe Riva, Matthew C. Costello, Claudia Repetto

**Affiliations:** 1 Applied Technology for Neuro-Psychology Lab, IRCCS Istituto Auxologico Italiano, Milan, Italy; 2 Humane Technology Lab, Università Cattolica del Sacro Cuore, Milan, Italy; 3 Department of Psychology, Università Cattolica del Sacro Cuore, Milan, Italy; 4 Department of Psychology, University of Hartford, Hartford, CT USA

**Keywords:** Frame of reference, Spatial cognition, Embodied cognition, Action language, Embodiment, Motor simulation

## Abstract

Growing evidence has revealed the crucial role of motor simulation and spatial perspective-taking in action language. However, there is still a lack of understanding of how motor and spatial processes interact when there are multiple actors involved, and if embodied processes are consistent across different cultures. To address this gap, we examined the interaction between motor simulation and spatial perspective-taking in action-sentences comprehension, along with the consistency of embodied processes across cultures. We collected data from Italian and US English speakers using an online sentence-picture verification task. The participants completed four conditions: two congruent (i.e., the participant is the agent in the sentence and the photo; the agent is someone else interacting with the participant in both the sentence and the picture) and two incongruent (i.e., the agents of the sentence and the picture do not match). The results show that when the perspective of the picture matched that described in the sentence-processing reaction times (RTs) were faster than in the incongruent conditions. In the congruent conditions where the agent is someone else, RTs were slower compared to the condition where the participant is the agent. This has been interpreted as claiming that motor simulation and perspective-taking are independent processes interacting during sentence comprehension (e.g., motor simulation is always run in the role of the agent, but we can adopt multiple perspectives depending on the pronouns and the contextual cues). Furthermore, Bayesian analysis provided evidence that embodied processing of action language entwines a common mechanism, suggesting cross-cultural consistency of embodied processes.

**Supplementary Information:**

The online version contains supplementary material available at 10.3758/s13421-023-01427-1.

## Introduction

The embodied cognition theory suggests the involvement of the sensorimotor systems during comprehension of action sentences (Barsalou, 2008; Beveridge & Pickering, 2013; Buccino et al., 2005; Glenberg & Kaschak, 2002; Hauk et al., 2004; Repetto et al., 2013; Tettamanti et al., 2005). Specifically, motor simulation (i.e., the simulation of the described action) and spatial perspective-taking (i.e., the simulation of the point of view assumed by the reader within the scene) seem to play a relevant role as fundamental processes involved in the understanding of action language (Beveridge & Pickering, 2013). However, the relationship between these two processes and their fundamental difference and relevance has not been fully understood yet (David et al., 2006; Decety & Sommerville, 2003; Ruby & Decety, 2001).

Strong positions within the embodied language approach conceive motor simulation as a mechanism linked to the role of the agent. Indeed, several pieces of evidence suggest that readers tend to simulate the agent’s actions through an embodiment process, and this is particularly true for action-related verbs (e.g., to peel) or sentences (e.g., “I hammer/he hammers”) involving only one character (Hauk et al., 2004; Repetto et al., 2013; Tettamanti et al., 2005; Tomasino et al., 2007). However, there are possible caveats regarding the motor simulation in the agent in the context of a two-character sentence. According to the spatial grounding hypothesis (SGH), motor simulations are grounded in the spatial context (Beveridge & Pickering, 2013). Specifically, the SGH posits that in complex sentences with many arguments, if there are minimal spatial cues sufficient to allow understanding the basic spatial relationships between the arguments, we can adopt different action perspectives other than the agent’s, including that of the receiver of an action (i.e., grammatical patient) or that of an external observer. Once we have assumed one spatial perspective, we simulate the action from that perspective. The minimum spatial information required is thought to be the participants in the action and some information about the spatial relations between them. For instance, the presence of a self-referential pronoun (“I” or “you” in the subject position, or “me” and “you” in other syntactic positions) should trigger the comprehender to assume the perspective consistent with the pronoun, centering the scene on one’s own body and positioning by default the rest of the participants in front of the reader (Beveridge & Pickering, 2013). Conversely, when no spatial cue is available, the reader cannot embody any of the participants' perspectives, therefore no motor simulation can take place (Gianelli et al., 2011; Greco, 2021; Papeo et al., 2011). For instance, Gianelli et al. (2011) showed that in the absence of spatial information, motor simulation of third-person sentences cannot occur, conversely when this information is presented, participants can simulate third-person agent sentences.

The idea that we can assume multiple perspectives in language understanding is consistent with the extant literature on spatial processing. In this regard, space can be coded according to two spatial frames of reference (Burgess, 2006, 2008): egocentric frame (body-dependent representation) and allocentric frame (body-independent representation). Hence, spatial frames of reference allow us to represent respectively the first-person perspective (our point of view) and the third-person perspective (someone else’s point of view) (Beveridge & Pickering, 2013; Tversky & Hard, 2009). The link between language and spatial frames of reference is supported by the activation of a widespread brain network recruited during both spatial navigation and a sentence-picture verification task (Vukovic & Shtyrov, 2017). Vukovic and Shtyrov showed that shared activity during navigation and sentence-picture verification tasks was found in the motor, extrastriate, premotor, and anterior cingulate regions. Motor and anterior cingulate areas are related to egocentric processing, whereas the extrastriate cortices involve the allocentric frame as well. In the context of single-character sentences, Brunyé et al. (2009), using a sentence-picture verification task, found that displaying on a PC screen sentences describing self-related actions (“I am slicing the tomato”/ “You are slicing the tomato”) facilitated the adoption of the first-person perspective (egocentric) compared to a third-person perspective (allocentric) when matching the sentences to photos. In contrast, sentences describing non-self-related actions (“He is slicing the tomato”) showed the opposite effect, with faster reaction times (RTs) for matching the sentence to pictures representing an allocentric point of view of the action. However, these results are questionable, as Brunyé and co-authors partially failed to replicate their previous findings. Specifically, in their second experiment (Brunyé et al., 2009), they demonstrated that the “I” pronoun did not facilitate egocentric perspective-taking compared to the “You” pronoun. In this second experiment, a brief context description was given prior to event sentences to enrich the descriptions, thus modulating the situated model of the described event. Again, a second study (Brunyé et al., 2016) showed that naturalistic narratives do not facilitate egocentric perspective-taking during the “I” compared with the “You” text. Moreover, Vukovic and Shtyrov (2017) showed that hearing “You” sentences facilitated first-person perspective photo processing compared to third-person perspective photo, whereas hearing “I” sentences displayed the opposite pattern.

These findings offer us a confusing set of conclusions. On the one hand, it seems clear that the process of understanding complex sentences calls into play both motor simulation and spatial perspective-taking, with different degrees of involvement depending on the task, the syntactic structure, and the specific content of the sentence. But on the other hand, the standing literature is currently unclear regarding if and how the processes of motor simulation and spatial perspective-taking interact in sentences with two characters (i.e., the participant and someone else) in which spatial cues are provided.

In addition, considering that our cognitive processes are not only embodied but also *situated* (Roth & Jornet, 2013), the role of the social context should be taken into account (Heeyon et al., 2010; Henrich et al., 2010a; 2010b; Leung et al., 2011). The relationship between language-driven motor simulation and social context has to date received limited empirical examination. Some authors (Heeyon et al., 2010) studied motor simulation in Korean subjects, and reported that cultural practices influence the action representation during language comprehension, and therefore motor simulation reflects the socially imposed constraints on action (Ghandhari et al., 2020; Henrich et al., 2010a; 2010b). More recently, Ghandhari and collaborators (Ghandhari et al., 2020) investigated the relationship between language and motor responses in two different linguistic cohorts, namely Italians and Persians. The different patterns of results for the two samples pointed to the need to consider cross-cultural differences when studying embodied mechanisms. Following this line of research, with the present study we aimed to investigate whether cultural differences can impact the processes of motor simulation and spatial perspective-taking differently in two populations, Italians and Americans. Indeed, a study on cross-cultural differences in egocentric and allocentric proclivity found that North Americans tend to adopt an allocentric frame of reference, whereas Europeans display a balanced use of egocentric and allocentric frames of reference (Goeke et al., 2015). This suggests that cultural background might shape the way we perceive and use spatial information.

In the current experiment, we used a two-agent sentence-picture verification task we developed following the procedure of Brunyè and colleagues (2009, 2016), and we tested it in two different languages (Italian and US English). Crucially, and differently from previous studies, we manipulated agency both in the sentence and in the photo. We created four experimental conditions: two congruent conditions and two incongruent conditions. In the first congruent condition, the participant is the agent in the sentence (i.e., “‘I’ am giving John the pen”) and photo (i.e., the photo depicts from an egocentric point of view the hands of the participant acting). In the second congruent condition, the agent is a third person (i.e., “John is giving ‘me’ the pen”) and in the photo, the agent is someone else in front of the participant (i.e., the photo depicts the participant’s egocentric point of view of the hands of someone else acting). In the incongruent conditions, there is an incongruency between the agent in the sentence and that in the image. According to the SGH, the presence of the self-referential pronoun should prompt comprehenders to assume the corresponding perspective, and as a consequence, they should simulate the action from that perspective. Therefore we expect that if simulation and perspective always match, the two congruent conditions should yield similar RTs since in both cases the pictures represent the perspective and the direction of the movement described in the sentence correctly. A converse possibility may arise if the strong view of embodiment is correct. According to this view (Decety, 2002), we always perform motor simulations as the agent of the action, even when the spatial perspective we assume is not that of the agent. As such, the condition in which I am the agent (with the pronoun “I” as a subject) should yield faster RTs compared to the condition in which the first-person pronoun occupies the thematic role of the receiver (with the pronoun “me”). In this latter case, motor simulation and spatial perspective would not overlap, and the picture would match only the perspective of the action but not its direction.

Lastly, our experimental design was specifically established to be cross-cultural, with separate cohorts of Italian and US English speakers. Under a strong embodiment hypothesis, we expect no differences between the two cultures/languages, suggesting that these processes reflect a common embodied mechanism. If culture plays a role, then we would expect that US English speakers, who seem to preferentially use an allocentric reference frame (Goeke et al., 2015), are less affected than Italians by the congruency of the spatial perspective.

## Method and materials

### Participants

Sixty-six Italian-speaking young adults (M_age_ = 24.95 years, SD_age_ = 2.8 years; 34 males; 58 right-handed) and 73 US English-speaking young adults (M_age_ = 25.69 years, SD_age_ = 4.2 years; 41 males; 64 right-handed) were recruited from Prolific (https://www.prolific.co/). Selection parameters of participants in the platform were set according to the self-reported age between 18 and 30 years, living in Italy/the USA, Italian/US English as a native language, self-reported absence of a history of psychiatric or neurological disorders (including language disorders), use of psychotropic drugs, and normal or corrected-to-normal vision. Participants were paid £11.88/h for performing the experiment (Italian median time = 15 m 09 s; USA median time = 15 m 24 s; time-limit of 25 min). To determine the sample size, we referred to the effect size found in a previous study that used a language-spatial perspective task similar to the one we developed (Brunyé et al., 2016). With a Cohen’s *d* of 0.35, a power of 0.8, and 80 target stimuli, the power analysis for a mixed-effects model (NCC design; i.e., participants are divided into two language conditions but every target stimulus is assessed under the two conditions) (Westfall et al., 2014) required a minimum total sample size of 117 participants (i.e., 58 approx. for each country). Participants were recruited online and gave their consent (Italian and English) to participate as approved by the Ethics Committees of the Catholic University of Milan.

### Sentence-picture verification task

A modified version of the sentence-picture verification task from Brunyé and co-authors (Brunyé et al., 2009) was created with Gorilla (Anwyl-Irvine et al., 2020), an online experiment builder for cognitive tasks. Forty sentences (20 in the first-person, e.g., “I am passing the tray to Marc”, and 20 in the third-person, e.g., “Paul is passing me the tray”, with the agent respectively being the first-person pronoun or a third-person subject) similar to Glenberg and Kaschak stimuli (Glenberg & Kaschak, 2002) were used (see Italian List in Online Supplementary Material (OSM) 1). In particular, sentences translated from US English to Italian were modified by replacing the “you” with the “I” pronoun and used in the present tense form as in the original task by Brunyé et al. (2009). In our experiment, we used part of the sentences used by Glenberg and Kaschak, in their seminal work on the action-sentence compatibility effect (2002). Although recent findings from a large multi-centric study (Morey et al., 2022) showed inconsistent results of the action-sentence compatibility effect (ACE), it is worth noticing that our experimental task/paradigm is different from the one typically used for investigating the ACE effect.

Forty wrists-to-hand photos depicting the 40 sentences were taken (accessories were removed; e.g., clocks or rings). The actor in the photo was right-handed and always performed the actions with the right hand. Six similar sentences and related photos were added to create the practice trials. Twenty photos represented actions from the camera's point of view (as if the viewer was acting), while in the other 20 images, the same actions had the opposite direction (someone else in front of the camera was acting). To create the pictures, the camera was placed either over the front of the actor with an elastic band or on a tripod in front of the actor. This set of stimuli was validated to test whether they portrayed the sentences depicted well. Fifty-four adults (age range 23–68 years) participated in the validation experiment (46 included after removing individuals with vision problems, self-reported language disorders, or spatial disorientation episodes). The sentences were presented one by one followed by the congruent image. Participants were asked to rate the extent to which the image was depicting the action of the sentence (“Does the photo match the sentence just shown?”; 0 = not at all to 10 = totally). Images with a total median score under 5 were shot again to improve hand position and/or gesture. All the photos are available via the Open Science Framework at https://osf.io/7s94v/.

For the sentence-picture verification task, each sentence was paired with both the congruent and the incongruent photo, so that in the congruent condition the agent was the same in the sentence and the photo, whereas in the incongruent pairs, the agent was not the same in the sentence and the photo. This resulted in a list of 80 target stimuli pairs (i.e., 80 trials) with four sentence-photo pair conditions (20 trials for each condition): 1_1 (the agent in the sentence and photo is the participant), 1_3 ( the agent in the sentence is the participant but the agent in the photo is someone else in front of the subject), 3_3 (the agent in the sentence is a third person and in the photo (s)he was someone in front of the participant), and 3_1 (the agent in the sentence is a third person but the agent in the photo is the participant). Following this design, conditions 1_1 and 3_3 are considered congruent since the agent in the sentence and picture matched, whereas conditions 1_3 and 3_1 are considered incongruent since the agent in the sentence and picture did not match.

In addition, we created a set of 20 sentence-photo pairs check-stimuli (i.e., 20 trials) in which the object held in the hand of the agent of the photo was not congruent with the object described in the sentence. From the original set of stimuli, 20 sentences were randomly picked a priori and matched with 20 photos where the object was not congruent. This resulted in five object-incongruent check trials for each condition (i.e., 1_1, 3_3, 1_3, 3_1). These check stimuli enabled us to control if participants read only the agent and/or the verb (i.e., without considering the second character) in the sentences to match the picture or read the whole sentence. Twenty-four additional pairs of stimuli (six for each condition), not included in the main task, were used as training trials for object congruent and incongruent conditions.

The sentences were translated by the Italian research team and then checked and corrected by the US research team in order to create the US English stimuli list (see USA List in OSM 1).

**Fig. 1:**
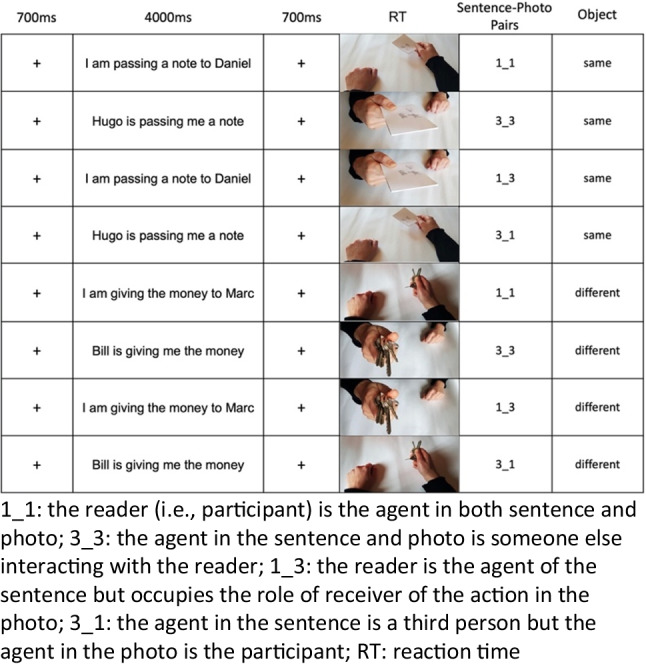
Procedure of the experiments. Example of agent and object congruent and incongruent conditions used in the experiment

In conclusion, we had 40 sentences translated into two languages and 40 photos used to create the experimental conditions and stimuli described above. Figure 1 shows the examples of each Sentence-Photo Pairs condition.

### Procedure

Once the experiment was published on the Prolific system, participants meeting the preselected inclusion/exclusion criteria could access the Gorilla link to start the task. After ticking the consent form (mandatory for proceeding further), demographic (i.e., age, sex, education, dominant hand) information was collected. After that, participants completed a practice block of 24 trials with the instructions (see below) with feedback for correct (green checkmark) and incorrect (red cross) responses before the actual task. In this way, participants could understand how to correctly match the sentence and the photo (i.e., by relying on the sentence-photo *agent* match-mismatch and also sentence-photo *object* match-mismatch).

For the main task, instructions (Italian or US English) were as follows: “You will see a series of sentences, each followed by an image. Your job is to understand the sentence and decide if the image correctly represents the sentence you just read. To answer, place the index of the right hand on the L key and the index of the left hand on the A key from the beginning of the experiment. Press A to indicate «YES – the image represents the sentence correctly» or Press L to indicate «NO – the image DOESN’T represent the sentence correctly». Speed matters – respond as quickly as you can while still being accurate.” Button order was counterbalanced across the participants. Each trial started with a fixation cross presented for 700 ms, then the sentence was shown for 4 s, followed by another 700-ms cross and the image. The stimuli pairs (i.e., sentence-photo pairs) list was randomized for each participant. RTs and accuracy rates were registered during the picture verification. Three attentional checks (i.e., find and click on the cat photo among eight images of dogs) across the 100 trials were placed. All participants passed the attention checks.

### Statistical analyses

The study is designed as a 2 x 2 x 2 between-within-subjects experiment, with one variable being Sentence Agent (first vs. third person; within-subjects variable), the second variable being the Photo Agent (the participant vs. someone else; within-subjects variable), and the third variable the Language of the sentences (Italian or US English; between-subjects variable). The combination of the two within-subjects variables yielded four within-subjects conditions (Sentence-Photo Pairs; labeled as follows: 1_1, 1_3, 3_1, 3_3) for each language. The first numbers represent the agent of the sentence (i.e., first or third) and the last numbers represent the agent of the photo (i.e., the participant or someone else).

All the analyses presented in this paper were performed by using R (R Core Team, 2014), version 3.6.3. Linear mixed-effects [*lme4* package (Bates et al., 2015)] ANOVAs were carried out with restricted maximum likelihood estimation (Luke, 2017). Single-term deletion was used to determine the significance of random effects (REs) in the model (Bates et al., 2015). All REs were set as having random intercepts, because all models failed to converge when allowing for random intercept and slope for these effects. Variance explained by RE on the dependent variable (RT) was provided by the intraclass correlation coefficient (ICC). The following formula was used in the R code: [picture verification RT ~ fixed effects + (1|participant ID) + (1|sentence ID) + (1|photo ID)]. The mixed-effects model diagnostic was assured for all models by visually checking residuals distribution and homoscedasticity. *Emmeans* package (Lenth, 2018) was used to analyze post hoc contrasts (1_1 vs. 3_3, 1_3 vs. 3_1, 1_1 vs. 1_3, and 3_3 vs. 3_1) with Bonferroni correction.

Benjamin and Berger’s (Benjamin & Berger, 2019) recommendations for p-value interpretation (p-value ≤ 0.005 “significant”; 0.005 < p-value < 0.05 “suggestive”) were followed. Effect size (η^2^_p_) was interpreted according to Richardson (2011) (small = 0.01, medium = 0.06, and large = 0.14), whereas Cohen’s d was interpreted according to Cohen’s rule of thumb (Sullivan & Feinn, 2012) (small = 0.2, medium = 0.5 and large = 0.8). The response variable in all the studies is always reported from the predicted values of the linear mixed-effects model. α level was set to 0.05.

## Results

Average accuracy before filtering (see below) for the actual task and the object congruent stimuli was 0.95 (SD = 0.08) and 0.9 (SD = 0.18) for the Italian and US English samples, respectively. For the object incongruent check stimuli, the average accuracy was 0.98 (SD = 0.03) and 0.94 (SD = 0.17) for the Italian and US English samples, respectively. The average accuracy for the stimuli pairs before filtering for the actual task and the object congruent stimuli was 0.95 (SD = 0.03) and 0.9 (SD = 0.06) for the Italian and US English sentences respectively. In OSM 1, Table 1 reports the training and actual task trials’ (without filtering) accuracy performance for each language.

Participants with an overall trial accuracy greater or equal to 80% were retained (3/66 removed for Italy; 10/73 removed for the USA). Thus, in the analysis, the number of included participants for each country was 63. No stimuli showed lower than 80% accuracy. We only analyzed the responses of the congruent object trials. Then RTs between 0 ms and 3,000 ms were included, so responses greater than 3,000 ms were removed, as in Brunyé and co-authors (2009). Only correct responses were included in the analyses and outliers were removed (within the Sentence-Photo Pairs and the two languages; 178 were removed for the Italian sample and 187 were removed for the US English sample) using the inter-quartile range method. Natural log transformation was used to improve skewness of the distribution.

T-tests were carried out to evaluate differences in age (p = 0.244) and years of education (p = 0.006) between the Italian and US English samples. Italian participants had a higher level of education (M = 15.51, SD = 2.33) than US English participants (M = 14.52, SD = 1.56). Chi-squared tests assessed any differences in the two samples regarding gender (p = 0.357) or dominant hand (p = 0.587).

### Random and fixed effects

In the first block of analysis, we put participants and stimuli (sentence and photo separately) as REs and the variables Sentence Agent, Photo Agent, and Language as fixed effects (2 x 2 x 2 levels between-within-subjects design). Participants and photos were found to be significant (participants p < 0.001; sentence p = 0.192; photo p < 0.001) and all REs represented 39.3% of the variance (participant ICC = 38.5%, sentence ICC = 0.3%, photo ICC = 0.5%) in the dependent variable (i.e., picture verification RT). This indicates that most of the variance was due to intra-individual variability.

**Fig. 2:**
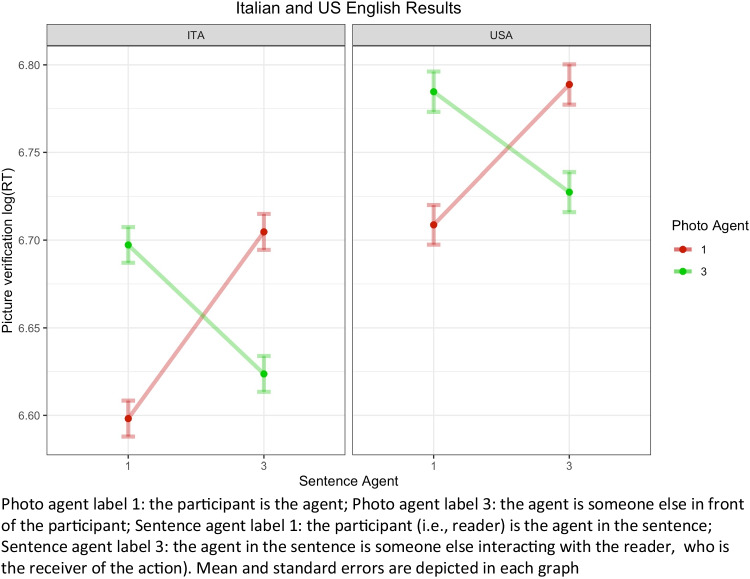
Interaction between motor simulation and spatial perspective-taking in the two cultures/languages

Findings indicated a significant interaction (Photo Agent by Sentence Agent by Language) (F_1, 9097_ = 5.22, p = 0.022, η^2^_p_ = 0, 95% CI [0, 0]). Other significant results were a main effect of Sentence Agent (F_1, 47_ = 4.54, p = 0.038, η^2^_p_ = 0.09, 95% CI [0, 0.27]), a main effect of Language (F_1, 126_ = 8.12, p = 0.005, η^2^_p_ = 0.06, 95% CI [0.01, 0.16], an interaction effect of Sentence Agent by Photo Agent (F_1, 9097_ = 248.45, p < 0.001, η^2^_p_ = 0.03, 95% CI [0.02, 0.03]. The main effect of Photo Agent (F_1, 25_ = 1.31, p = 0.263), the interaction effect of Sentence Agent by Language (F_1, 47_= 0.34, p = 0.561), and Photo Agent by Language (F_1, 9097_ = 0.20, p = 0.654) were not significant. Figure 2 shows the interaction divided by language.

As the Photo Agent × Sentence Agent × Language interaction was significant, we proceeded with the second set of analyses to explore the contrasts of interest in relation to language. We put participants and stimuli (sentences and photos) as REs with random intercept and the variable Sentence-Photo Pairs (four levels within-subjects design) and Language (two between levels) as a fixed effect. Participants and photos were found to be significant (participants p < 0.001; sentence p = 0.2; photo p < 0.001) and all REs represented 39.3% (ICC) of the variance in the RTs (participant ICC = 38.5%, sentence ICC = 0.3%, photo ICC = 0.5%). Again, most of the REs variability on the RTs was due to participants rather than stimuli pairs.

Results indicated a significant effect of the Sentence-Photo Pairs (F_3, 125_ = 84.81, p < 0.001, η^2^_p_ = 0.67, 95% CI [0.58, 0.74]). In addition, we found a main effect of Language (F_1, 126_ = 8.12, p = 0.005, η^2^_p_ = 0.06, 95% CI [0.01, 0.16]). In particular, Italian participants were faster (M = 6.66, SD = 0.18) than US English participants (M = 6.75, SD = 0.2) regardless of the Sentence-Photo Pairs. Crucially, the interaction between Sentence-Photo Pairs and Language was no longer significant (F_3, 276_ = 1.92, p = 0.126).

**Fig. 3:**
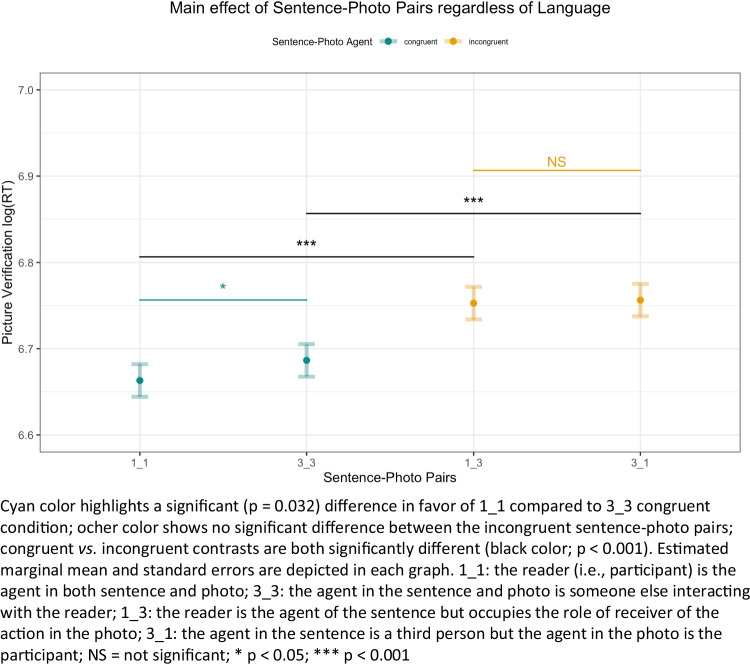
Summary of the significant effects of study

As hypothesized, regardless of the language of the participants, planned contrast showed a suggestive difference between the congruent conditions 1_1 and 3_3 (t_72_ = -2.18, p = 0.032, d = -0.26, 95% CI [-0.49, -0.02]). Specifically, when the agent was the participant in both, the sentence and image verification times were faster (M_log(1_1)_ = 6.66, SE_log(1_1)_ = 0.02) than when the agent was someone else in the sentence and photo (M_log(3_3)_ = 6.68, SE_log(3_3)_ = 0.02). In particular, 60.32% of the participants showed lower RTs for the 1_1 compared to the 3_3 condition. Moreover, we found significant differences between the congruent and incongruent conditions. In particular, contrasts 1_1 versus 1_3 (t_57_ = -8.99, p < 0.001, d = -1.19, 95% CI [-1.52, -0.85]) and 3_3 versus 3_1 (t_57_ = -6.99, p < 0.001, d = -0.93, 95% CI [-1.23, -0.61]). In the congruent condition 1_1, verification times were faster (M_log(1_1)_ = 6.66, SE_log(1_1)_ = 0.02) than in the incongruent condition 1_3 (M_log(1_3)_ = 6.75, SE_log(1_3)_ = 0.02); similarly in the congruent condition 3_3, verification times were faster (M_log(3_3)_ = 6.68, SE_log(3_3)_ = 0.02) than in the incongruent condition 3_1 (M_log(3_1)_ = 6.76, SE_log(3_1)_ = 0.02). There were no significative differences between the incongruent condition 1_3 versus 3_1 (t_72_ = -0.33, p = 0.739), suggesting that mismatching conditions have a comparable effect on verification RTs. Figure 3 represents the effects found regardless of the language of the samples. Table 2 in OSM 1 provides untransformed RTs of the four conditions.

To control for a potential confounding effect of the participant handedness, we added this variable to the model, and the main effect of Sentence-Photo Pairs was still significant (F_3, 125_ = 84.81, p < 0.001, η^2^_p_ = 0.67, 95% CI [0.58, 0.74]). The main effect of Language (F_3, 120_ = 1.69, p = 0.196), the handedness covariate (F_3, 120_ = 0.586, p = 0.625), and the interaction Sentence-Photo Pairs by Language (F_3, 277_ = 1.92, p = 0.126) were not significant. Thus, the final model was the one with only Sentence-Photo Pairs and Language as fixed effects.

### Cross-cultural Bayesian evidence

To support the validity of our findings at a cross-cultural level, we used the Bayesian statistics Bayes factor bound (BFB) computation. Jeffreys’ rule of thumb for BFB interpretation was used (Ly et al., 2016). Evidence from the data in favor of H_1_ relative to H_0_ (BFB), odds in favor of H_1_ relative to H_0_ prior to seeing the data, and “post-experimental odds” (odds given the data) combined with prior odds of H_1_ to H_0_ (set 1:1 as we do not have prior odds in favor of a specific hypothesis) were computed as suggested (Benjamin & Berger, 2019). The use of the BFB provides complementary information to p-values, which helps to answer the question “How strongly does the evidence favor the alternative hypothesis relative to the null hypothesis?”, which cannot be directly answered by using p-value alone (Benjamin & Berger, 2019).

To test the hypothesis of motor simulation in the agent, we used post hoc contrasts (1_1 vs. 3_3) p-values with Bonferroni correction. To test the hypothesis that conditions 1_3 and 3_1 require both motor simulation and spatial perspective-taking in the agent, the relevant contrasts’ p-values with Bonferroni correction were used (as p-values are all < 0.001, we used 0.001 for the computation). Regarding the motor simulation (1_1 vs. 3_3) in the agent, results showed substantial evidence (1.10 < natural log of BFB < 2.30; Ly et al., 2016) in favor of H_1_ and a probability of 19% of H_0_ being true prior to seeing the data. In addition, given flat prior odds, H_1_ is given approximately 4 to 1.

For the embodiment in the agent through motor simulation and spatial perspective-taking (1_1 vs. 1_3 and 3_3 vs. 3_1), findings demonstrate very strong evidence (BFB > 3.4; Ly et al., 2016) in favor of H_1_ for both conditions and a probability of 2% of H_0_ being true in all conditions. In addition, given flat prior odds, H_1_ is given approximately 53 to 1 in both conditions.

Importantly, evidence in favor of a difference among the Sentence-Photo Pairs conditions due to Language is null (log of BFB = 0.34). The probability of H_1_ (i.e., no interaction) being true prior to seeing the data is 59%, and given the flat prior odds (1:1), H_1_ is given approximately 1 to 1. Despite the fact that the null hypothesis cannot be completely ruled out, our results point in the direction of cross-cultural Bayesian evidence of our hypotheses. Table 1 shows the BFB, the odds (Pr^*U*^ (*H*_1_|*p*)) for H_1_ to H_0,_ and the post-experimental odds.

**Table 1:** Cross-cultural Bayesian evidence

Effect	Hypotheses	p-value	log (BFB)	Pr^*U*^ (*H*_1_|*p*)	Post-experimental odds
Culture	H_1_: Sentence-Photo Pairs by Language interaction H_0_: absence of interaction	0.126	0.34	0.59	1.14:1
Agent motor embodiment in the two cultures	H_1_:μ_1_1_ ≠ μ_3_3_ H_0_:μ_1_1_ = μ_3_3_	0.032	1.21	0.77	3.35:1
Agent motor and spatial embodiment in the two cultures	H_1_:μ_1_1_ ≠ μ_1_3_ H_0_:μ_1_1_ = μ_1_3_	< 0.001	> 3.97	0.98	53.42:1
	H_1_:μ_3_3_ ≠ μ_3_1_ H_0_:μ_3_3_ = μ_3_1_	< 0.001	> 3.97	0.98	53.42:1

1_1: the reader (i.e., participant) is the agent in both sentence and photo; 3_3: the agent in the sentence and photo is someone else interacting with the reader; 1_3: the reader is the agent of the sentence but occupies the role of receiver of the action in the photo; 3_1: the agent in the sentence is a third person but the agent in the photo is the participant.

*BFB* Bayes factor bound

Pr^*U*^ (*H*_1_|*p*): odd for H_1_ to H_0_ prior to seeing the data; BFB between 0 and 1.10 represents null evidence in favor of H_1_; BFB between 1.10 and 2.30 represents substantial evidence in favor of H_1_; BFB > 3.4 is indicative of very strong evidence

## Discussion

In this study, we sought to explore the interplay between motor simulation and spatial perspective-taking processes in sentences involving two actors through an action sentence-picture verification task. We found that the congruent condition, where the participant is the agent in both sentence and picture (1_1), is processed faster compared to the other three conditions (3_3, 1_3, and 3_1). The congruent condition where the agent is someone else in both sentence and photo (3_3) is processed slower compared to 1_1, but faster than the incongruent conditions (1_3 and 3_1). In addition, the incongruent conditions are processed slower than the congruent pairs and are not different from each other. Lastly, we demonstrated that our findings are cross-cultural and occur in at least two different languages, indicating possibly common embodied processing.

The crucial comparison for our study was between 1_1 and 3_3. The fact that 1_1 resulted in faster responses than 3_3 seems to support the hypothesis that the motor simulation takes place in the agent. Indeed, in the 1_1 condition, the subject of the sentence is the agent of the action, therefore motor simulation and spatial perspectives overlap. This is consistent with previous research on self-consciousness that points to the pivotal role of the first-person agency and spatial perspective in our phenomenology and psychology of the self (Blanke, 2012; Eich et al., 2009; Tversky & Hard, 2009; Vogeley & Fink, 2003). In addition, this finding is in line with Brunyé and co-authors (Brunyé et al., 2009), where first-person (“I”) sentences led to the adoption of the first-person (egocentric) perspective.

On the other hand, in the 3_3 condition, we suppose that the reader assumes the spatial perspective of the receiver, prompted by the referential “me” pronoun (see SGH), but at the same time, the reader runs a motor simulation as if he/she was the agent (strong theory on embodiment; e.g., Decety, 2002). When matching the sentence to the picture, a short delay was registered since the picture corresponds to the sentence in the spatial perspective, but the displayed movement is in the opposite direction with respect to the agency. It is possible that the activation of the motor and premotor cortex, which has been identified as proof of motor simulation during sentence processing (Hauk et al., 2004; Tettamanti et al., 2005), slightly interferes with the movement observed in the picture. Such interference effects between real and simulated movements have already been described in language tasks. For example, some studies have found a selective interference between action words and action execution involving the same effector (Dalla Volta et al., 2009; Liepelt et al., 2012; Mirabella et al., 2012; Nazir et al., 2008; Sato et al., 2008). Our findings extended SGH findings, demonstrating that the reader assumes the spatial perspective of the receiver, consistently with the syntactic role occupied by the self-referential pronoun, but also simulates the action carried out by the agent in the photo.

Following this line of reasoning, slower reaction times in 1_3 conditions compared to the congruent conditions are accounted for by the violation of both action direction (simulation) and spatial perspective represented in the picture when confronted with the sentence. In the sentence, the reader assumes the agent’s perspective because of the pronoun “I” in the subject position, and simulates the action as such; in the picture, the position of the reader is displayed as the receiver, and the movement is depicted in the correspondent direction. The condition 3_1, however, is unexpectedly as slow as the 1_3 condition, even if in this case the match between sentence and picture should imply only one violation (i.e., the perspective), making it more similar to the 3_3 than to the 1_3 condition. One possible explanation is related to the type of task employed. Considering that the sentence-picture verification task is prominently visual in nature, and therefore the spatial perspective violation may have a greater impact than the motor violation. If this is true, we should expect an opposite pattern of results in a motor task (i.e., 3_3 as slower as 1_3). The task demands have been identified as a key factor to account for contrasting results: for example, the emphasis on the action execution or the imagery of the action can explain inconsistencies in perspective-taking (Pecher et al., 2009; Zwaan & Taylor, 2006). Future studies could specifically address this issue by comparing different kinds of tasks directly.

Finally, our findings suggest that embodied simulations of the agent’s action and spatial perspective are not necessarily tied to cultural context (Goeke et al., 2015; Henrich et al., 2010b), but seem to be shared in at least Italian and US English. To our knowledge, no group has assessed embodied linguistic effects within the context of Italian and US English. The striking similarities between our two samples might support the concept of cross-cultural embodied processes (cf., Sinha & Kristine, 2001). In other words, the cultural difference in the preferential use of allocentric versus egocentric frames of reference did not affect motor simulation and spatial perspective taking during language comprehension, pointing to shared mechanisms of language embodiment. Although our results are supportive of such a possibility, an obvious limitation is that our samples assessed only two language cohorts. Future research could administer our testing paradigm to other languages, targeting populations with more diverse cultures (i.e., not Western cultures).

Despite encouraging results, our study has limitations, as we did not consider other psychological confounding variables that could come into play (i.e., accounting for low effect size), like executive functions, egocentric/allocentric spatial preferences, or empathy measures (Brunyé et al., 2016; Gardner et al., 2013; Vukovic & Williams, 2015). In addition, we used online testing, and in-person data collection should replicate these findings. Lastly, we acknowledge that the sentences being used do not grasp the complexity of everyday life narrative comprehension. As stated in a recent consensus paper (Ibanez et al., 2022), it is crucial to consider the potential effect of context (e.g., laboratory vs. ecological setting, culture, or cross-cultural differences) on the simulation of language. In addition, we showed that only 60.32% of the participants showed faster 1_1 versus 3_3 mean RTs. Again, individual differences (Ibanez et al., 2022) are critical when studying embodied language simulation and comprehension. Future studies should consider how these individual differences might impact action language simulation.

To conclude, this is the first study to our knowledge that explored the relation between motor simulation and spatial perspective-taking in two-character action-related sentences. We showed the two mechanisms are likely, at least partially, independent, and that motor simulation can occur separately from the perspective assumed when we are not the agent of the sentence. Further, we confirmed that we can assume multiple perspectives during comprehension and that the presence of a self-referential pronoun can prompt us to select the specific perspective among all the possible alternatives allowed by the sentence. Lastly, the common results from our Italian and English samples offer evidence of a cross-cultural and potentially universal embodied language effect. Our findings should be supported by imaging studies, which could show whether the activation of motor and premotor cortices arises using sentences with a third person as a subject and the self-referential pronoun as a receiver. Furthermore, future studies could investigate abnormal agent embodiment in neurodegenerative and psychiatric diseases in which social and/or spatial domains are compromised to better understand the interplay between action language understanding and spatial information (Buckner et al., 2008; Kemp et al., 2012; Serino et al., 2014; Tuena et al., 2021).

### Supplementary Information

ESM 1 (DOCX 19 kb)

## Data Availability

Data, stimuli (photos), and codes of both experiments are available via the Open Science Framework at: https://osf.io/7s94v/. Sentences are available in the Online Supplementary Material 1. This study was not pre-registered.
