# Supplementary material for: The interaction between motor simulation and spatial perspective-taking in action language: a cross-cultural study
Source: Mem Cognit. 2023 May 19;51(8):1870–80. doi: 10.3758/s13421-023-01427-1 (PMC10638199; doi:10.3758/s13421-023-01427-1)
Supplement: Supplementary file 1 — (DOCX 19 kb) [file 13421_2023_1427_MOESM1_ESM.docx]

Supplementary Material 1

Table 1 Accuracy for the conditions

| Sentence-Photo Pairs | Object | Average accuracy (SD) | Language | Task |
| --- | --- | --- | --- | --- |
| 1_1 | same | 0.92 (0.16) | Italian | training |
| 1_1 | different | 0.94 (0.14) | Italian | training |
| 1_1 | same | 0.96 (0.05) | Italian | actual |
| 1_1 | different | 0.97 (0.09) | Italian | actual |
| 3_3 | same | 0.93 (0.14) | Italian | training |
| 3_3 | different | 0.98 (0.1) | Italian | training |
| 3_3 | same | 0.97 (0.05) | Italian | actual |
| 3_3 | different | 0.98 (0.07) | Italian | actual |
| 1_3 | same | 0.7 (0.26) | Italian | training |
| 1_3 | different | 0.99 (0.04) | Italian | training |
| 1_3 | same | 0.95 (0.15) | Italian | actual |
| 1_3 | different | 0.99 (0.09) | Italian | actual |
| 3_1 | same | 0.73 (0.29) | Italian | training |
| 3_1 | different | 0.98 (0.1) | Italian | training |
| 3_1 | same | 0.93 (0.16) | Italian | training |
| 3_1 | different | 0.99 (0.04) | Italian | training |
| 1_1 | same | 0.88 (0.19) | US English | training |
| 1_1 | different | 0.91 (0.2) | US English | training |
| 1_1 | same | 0.95 (0.13) | US English | actual |
| 1_1 | different | 0.93 (0.18) | US English | actual |
| 3_3 | same | 0.87 (0.23) | US English | training |
| 3_3 | different | 0.9 (0.22) | US English | training |
| 3_3 | same | 0.95 (0.13) | US English | actual |
| 3_3 | different | 0.91 (0.19) | US English | actual |
| 1_3 | same | 0.60 (0.34) | US English | training |
| 1_3 | different | 0.93 (0.19) | US English | training |
| 1_3 | same | 0.85 (0.28) | US English | actual |
| 1_3 | different | 0.97 (0.17) | US English | actual |
| 3_1 | same | 0.63 (0.35) | US English | training |
| 3_1 | different | 0.94 (0.16) | US English | training |
| 3_1 | same | 0.85 (0.27) | US English | actual |
| 3_1 | different | 0.95 (0.16) | US English | actual |

Table 2 Untransformed raw RT

| Condition | N | Mean RT | SD | Min | Max |
| --- | --- | --- | --- | --- | --- |
| 1_1 | 2341 | 814.72 | 264.55 | 353.4 | 1727.4 |
| 3_3 | 2334 | 834.21 | 270.46 | 307.7 | 1711.4 |
| 1_3 | 2312 | 888.41 | 285.14 | 362 | 1862 |
| 3_1 | 2323 | 894.41 | 289.55 | 391.3 | 1869.8 |

| **List Sentence Exp 1** | **List Sentence Exp 2** | **Perspective** |
| --- | --- | --- |
| Luca mi sta tirando un elastico | Alex is shooting the rubber band at me | Third-person |
| Sto tirando l'elastico a Sofia | I am shooting the rubber band at Luke | First-person |
| Sara mi sta versando l'acqua | Sarah is pouring me a cup of water | Third-person |
| Sto versando dell'acqua a mio padre | I am pouring a cup of water for my father | First-person |
| Marco mi sta porgendo la palla da calcio | Marc is passing me the soccer ball | Third-person |
| Sto porgendo a Mario la palla da calcio | I am passing the soccer ball to Marc | First-person |
| Mario mi sta passando un bigliettino | Hugo is passing me a note | Third-person |
| Sto passando un bigliettino a Mario | I am passing a note to Daniel | First-person |
| Carlo mi sta restituendo la biglia | Charles is giving me back the marble | Third-person |
| Sto restituendo la biglia a Sandro | I am giving the marble back to Alex | First-person |
| Emma mi sta regalando la palla di carta | Emma is giving me a paper ball | Third-person |
| Sto regalando la palla di carta a Nadia | I am giving a paper ball to Ella | First-person |
| Giulia mi sta prestando la penna | Mary is lending me a pen | Third-person |
| Sto prestando la penna a Laura | I am lending a pen to Jasmine | First-person |
| Paolo mi sta porgendo il vassoio | Paul is passing me the tray | Third-person |
| Sto porgendo il vassoio a Marco | I am passing the tray to Marc | First-person |
| Chiara mi sta dando un gelato | Claire is giving me ice-cream | Third-person |
| Sto dando un gelato a Claudia | I am giving ice-cream to Celine | First-person |
| Claudio mi sta consegnando il quaderno | Claire is handing me the notebook | Third-person |
| Sto consegnando il quaderno a Sara | I am handing the notebook to Sarah | First-person |
| Livio mi sta passando la palla | Matthew is passing me the ball | Third-person |
| Sto passando la palla a Luca | I am passing the ball to Luke | First-person |
| Claudio mi sta passando il pupazzo | Charles is passing me the puppet | Third-person |
| Sto passando il pupazzo ad Anna | I am passing the puppet to Hanna | First-person |
| Bruno mi sta dando le carte | Jack is dealing me the cards | Third-person |
| Sto dando le carte a Laura | I am dealing the cards to Peter | First-person |
| Maria mi sta affidando le chiavi | Mary is handing me the keys | Third-person |
| Sto affidando le chiavi a Sandra | I am handing the keys to Sandra | First-person |
| Luca mi sta distribuendo le razioni | Laura is breaking off a piece of chocolate for me | Third-person |
| Sto distribuendo le razioni a Dario | I am breaking off a piece of chocolate for Bill | First-person |
| Paola mi sta rendendo la macchinina | Paula is handing me the little car | Third-person |
| Sto rendendo la macchinina ad Alessia | I am handing the little car to Alexia | First-person |
| Fulvio mi sta sganciando i soldi | Bill is giving me the money | Third-person |
| Sto sganciando i soldi a Marco | I am giving the money to Marc | First-person |
| Anna mi sta donando del denaro | Hanna is donating money to me | Third-person |
| Sto donando del denaro a Laura | I am donating money to Laura | First-person |
| Alessia mi sta premiando con una medaglia | Alexia is awarding me a medal | Third-person |
| Sto premiando Sonia con una medaglia | I am awarding Bobbie a medal | First-person |
| Bruno mi sta consegnando la pizza | Irwin is delivering a pizza to me | Third-person |
| Sto consegnando la pizza a Dario | I am delivering a pizza to Bill | First-person |
